# Development of a newborn screening tool for mucopolysaccharidosis type I based on bivariate normal limits: Using glycosaminoglycan and alpha‐L‐iduronidase determinations on dried blood spots to predict symptoms

**DOI:** 10.1002/jmd2.12093

**Published:** 2020-02-10

**Authors:** Thomas J. Langan, Kabir Jalal, Amy L. Barczykowski, Randy L. Carter, Molly Stapleton, Kenji Orii, Toshiyuki Fukao, Hironori Kobayashi, Seiji Yamaguchi, Shunji Tomatsu

**Affiliations:** ^1^ Department of Neurology, School of Medicine and Biomedical Sciences University at Buffalo Buffalo New York; ^2^ Department of Biostatistics, Population Health Observatory, School of Public Health and Health Professions University at Buffalo Buffalo New York; ^3^ Skeletal Dysplasia Research Lab, Nemours/Alfred I. DuPont Hospital for Children Wilmington Delaware; ^4^ Department of Biological Sciences University of Delaware Newark Delaware; ^5^ Department of Pediatrics, Graduate School of Medicine Gifu University Gifu Japan; ^6^ Department of Pediatrics Shimane University Shimane Japan; ^7^ Department of Pediatrics Thomas Jefferson University Philadelphia Pennsylvania

**Keywords:** alpha‐L‐iduronidase, biomarkers, glycosaminoglycans, heparan, mucopolysaccharidosis type I, newborn screening, sulfate

## Abstract

**Purpose:**

Current newborn screening (NBS) for mucopolysaccharidosis type I (MPSI) has very high false positive rates and low positive predictive values (PPVs). To improve the accuracy of presymptomatic prediction for MPSI, we propose an NBS tool based on known biomarkers, alpha‐L‐iduronidase enzyme activity (IDUA) and level of the glycosaminoglycan (GAG) heparan sulfate (HS).

**Methods:**

We developed the NBS tool using measures from dried blood spots (DBS) of 5000 normal newborns from Gifu Prefecture, Japan. The tool's predictive accuracy was tested on the newborn DBS from these infants and from seven patients who were known to have early‐onset MPSI (Hurler's syndrome). Bivariate analyses of the standardized natural logarithms of IDUA and HS levels were employed to develop the tool.

**Results:**

Every case of early‐onset MPSI was predicted correctly by the tool. No normal newborn was incorrectly identified as having early‐onset MPSI, whereas 12 normal newborns were so incorrectly identified by the Gifu NBS protocol. The PPV was estimated to be 99.9%.

**Conclusions:**

Bivariate analysis of IDUA with HS in newborn DBS can accurately predict early MPSI symptoms, control false positive rates, and enhance presymptomatic treatment. This bivariate analysis‐based approach, which was developed for Krabbe disease, can be extended to additional screened disorders.

**Synopsis**

A bivariate normal limits‐based approach to newborn screening for mucopolysaccaridosis type 1 can potentially eliminate false positive screens and enable early identification and treatment of affected infants before symptoms emerge.

## 1 INTRODUCTION

The mucopolysaccharidoses (MPS) are caused by deficiencies of lysosomal enzymes, which cause accumulation of the complex polysaccharides known as glycosaminoglycans (GAGs).^1^, ^2^ Deposition occurs in brain, visceral organs, muscle, and bone with devastating clinical effects. These include, bone deformity, growth failure, coarse facies, developmental delays, intellectual regression, visceromegaly, muscle weakness, and cardiac problems.^1^, ^2^

Mucopolysacharidosis type 1 (MPSI) (or Hurler syndrome, OMIM# 607014)^1^, ^2^ has a variable age of onset, although early‐onset (ie, nonattenuated), severe phenotypes emerge in early infancy.^1^, ^2^ Assays of the enzyme deficient in MPSI has been adapted to newborn blood spots.^2^, ^3^, ^4^ Treatment for MPSI is available through enzyme replacement therapy and stem cell transplantation.^5^, ^6^, ^7^, ^8^, ^9^

It has consequently been suggested that this conditions is amenable to newborn screening (NBS).^5^, ^6^, ^7^, ^8^, ^9^ NBS pilot studies have been initiated in the United States as well as other countries.^2^, ^3^ In addition, MPSI was added in 2016 to the recommended universal screening panel in the United States.^2^, ^10^ Thus live screening for MPSI is currently being performed in 13 US states.^2^, ^10^

The newborn pilot studies that preceded live screening demonstrated that the early‐onset, nonattenuated phenotype of MPSI can be identified successfully through NBS. The daunting problem of identifying the severe late onset phenotypes must however be noted, which may require genotyping after NBS. In addition, these pilot studies were plagued by excessive false positives. This problem can be quantified by comparison of the positive predictive values (PPVs) of the respective screening protocols; PPV is defined as the ratio of true positive screens to all positive call outs.^11^ In the absence of true positive cases in a given sample, PPV may be estimated by use of a disease prevalence rate, sensitivity, and false positive rate. Our prior research developed methods to verify that a targeted false positive rate is achieved given normality assumptions.^12^, ^13^

Pilot studies were, for example, performed with dried blood spots (DBS) in Taiwan^14^ and in Italy.^15^ The PPV values for MPSI were 26.7% and 7.7%, respectively. In the United States, pilot studies conducted, for example, in Kentucky,^16^ Illinois,^17^ Missouri^18^ and New York,^19^ respectively, yielded PPVs of 50%, 0.46%, 3.0%, and 0% for MPSI NBS.

Thus, low PPVs support the conclusion that false positive rates are excessive in MPSI NBS. The resulting uncertainty as to whether symptoms are imminent will engender a diagnostic odyssey comprised of significant anxiety for families and medical professionals alike.^20^

We developed a NBS diagnostic approach to Krabbe disease (KD) employing a bivariate analysis of biomarkers from 18 known cases that resulted in a very high PPV of 98.5%.^12^, ^13^ A multi‐tiered screening platform also reported a high PPV for KD, since their single known case was correctly diagnosed.^16^ But that study yielded an overall PPV of 80%^16^ for screening of lysosomal disorders, suggesting that the bivariate analyses may enable relative enhancement of diagnostic accuracy.^12^, ^13^ We present here evidence that the bivariate approach is also applicable to MPSI, and may remedy the diagnostic odyssey inherent to NBS for this disorder.

## 2 MATERIALS AND METHODS

### 2.1 Bloodspots and patient data

Five thousand newborn spots were acquired prospectively by Gifu Prefecture, Japan.^4^ Twelve of these newborn infants had been predicted to become MPSI patients by the Gifu NBS protocol, but had not developed MPS symptoms after 2 years of follow‐up.^2^, ^4^ These 12 were considered to have a “pseudo‐deficiency,” with low alpha‐L‐iduronidase (IDUA) enzyme but no symptoms.^2^, ^4^, ^18^ Separately, newborn DBS were obtained retrospectively for seven known nonattenuated MPSI patients.^4^ These cases were not from the Gifu MPSI pilot program, and repeat analyses for them are not available. The Gifu program includes prospective measurement of IDUA enzyme and several GAGs but not genetic analyses.

### 2.2 Assays of GAGs and IDUA

GAGs were assayed on newborn DBS as described previously using liquid chromatography and tandem mass spectroscopy.^4^, ^21^ In a pilot study, it was found that measurements of control samples had unacceptably high standard deviations, which were attributed to potential elevations of GAGs in other non‐MPS conditions, a limited sample size derivable from these DBS and the inherent complexity of the assay.^4^

Hence assay of the IDUA enzyme still remains the gold standard for first tier NBS for MPS, although GAG determination can potentially assist phenotypic prediction.^2^, ^19^ Among the GAGs assayed and known to be elevated in MPSI,^2^, ^3^, ^4^ heparan ΔDi‐NS (2‐deoxy‐2‐sulfamino 4‐O‐[4‐deoxy‐α‐L‐*threo*‐hex‐4‐enopyranosyluronic acid]‐D‐glucose) (HS) was demonstrated (results not shown) to provide the most consistent results. Thus, HS was considered together with IDUA for the current bivariate normal limits (BVNL) approach. We acknowledge, that prospective testing of the proposed tool should include additional GAGs elevated in MPSI.^2^, ^3^, ^4^

In the Gifu pilot, the average IDUA and HS levels in the normal newborn DBS were 70.57 nmol/mg/hour and 45.12 ng/mL, respectively. The cut‐off values chosen to eliminate false negatives were under 16.68 for IDUA and over 90.00 for HS.

### 2.3 Statistical analyses

#### 2.3.1 BVNL construction

Development of (BVNL)‐based NBS tools for MPSI followed that of tools for KD screening^12^, ^13^ under a working assumption that the pairs of biomarkers involved have a bivariate normal distribution. Probability plots of the natural logarithms of HS with IDUA were generated from the normative sample of the 5000 normal newborns. The points in each plot followed an approximate straight line pattern, consistent with normally distributed variables and bivariate normality.

Multivariate normal distribution theory^22^ and associated formulas for (1 − *p*) 100% prediction regions^23^ were employed, where *p* represents the portion of future observations expected to fall outside the prediction region. Thresholds of −3.62 and 1.90 for standardized log_e_(IDUA) and log_e_(HS), were chosen, respectively. Prediction regions (ellipses) for univariately standardized natural logarithms of HS and IDUA measurements of the 5000 normal newborns were calculated with *p =* 10^−7^. Tolerance for false positive rate (*fp*; 1 − specificity dictates thresholds for “low” IDUA, “high” HS values, and choice of *p*. These values were chosen to control the false positive rate at *fp* = 10^−8^.

The decision rule of the BVNL test was based on IDUA and HS and states that an infant tests positive for MPSI only if standardized log_e_(IDUA) < −3.62, standardized log_e_(HS) > 1.90, and the point representing these values falls outside of the (1‐10^−7^) 100% prediction ellipse for standardized log_e_(IDUA) and log_e_(HS).

#### 2.3.2 Univariate threshold‐based tests

NBS programs typically rely on univariate thresholds for enzyme and biomarker measures.^12^, ^16^ Thus, we define the following NBS classification rules:

1. IDUA‐based univariate rule—Classify as test positive if standardized log_e_(IDUA) < −3.62. Otherwise, classify as negative;
2. HS‐based univariate rule—Classify as test positive if standardized log_e_(HS) > 1.90. Otherwise, classify as negative;
3. IDUA, HS joint univariate threshold based rule—Classify as test positive if standardized log_e_(IDUA) < −3.62 and standardized log_e_(HS) > 1.90. Otherwise classify as negative.

Our BVNL‐based tool is compared with respect to sensitivity, specificity, false positive rate, and false negative rate.

#### 2.3.3 Estimation of sensitivity, false positive rate, and PPV

Under our working assumptions, these choices of thresholds and *fp* would in theory control the false positive rate of the BVNL‐based tool at approximately 10^−8^ and thus control specificity at (110^‐8^), or 0.99999999. For comparison, we estimated the observed false positive rate as the proportion of normal newborns is that tested positive of and the observed specificity as 1 minus this estimated false positive rate. Sensitivity was estimated as the proportion of the seven known MPSI cases who had a positive NBS test result and the observed false negative rate as 1 minus this estimated sensitivity. False positive rate, specificity, sensitivity, and false negative rate were estimated for each of the classification rules defined above in the univariate threshold‐based tests subsection.

PPV is usually estimated as the ratio of diseased individuals to those with a positive test. Alternatively, PPV is estimated using the previously established formula.

$$\mathrm{PPV}=\text{Sens}\times \text{Prev}/\left(\text{Sens}\times \text{Prev}+\mathrm{FP}\times \left[1-\text{Prev}\right]\right),$$

where Sens, Prev, and FP are the sensitivity of the diagnostic test, prevalence of disease, and false positive rate of the test, respectively.^24^ In this study, in order to obtain an estimate of the PPV for the BVNL tool we substitute into this formula an estimate of Prev (ie, 10^5^) from the literature,^25^, ^26^ the theoretically expected false positive rate of the BVNL‐based tools (*fp* = 10^−8^), and an estimate of sensitivity based on test results for our seven cases of MPSI.

#### 2.3.4 Simulation study

Given our choices of thresholds and targeted false positive rate (*fp* = 10^−8^), one falsely predicted MPS I case is expected when testing 100 million normal newborns. In addition, because any false positive result from a BVNL test will also be a false positive by associated univariate threshold‐based tests but not vice versa, we know theoretically that the false positive rate of the BVNL test will be lower than that of univariate tests but we do not know how much lower. We performed a simulation study to compare BVNL‐based tests to three alternative tests based on the three univariate noted above.

The bivariate distribution of logeIDUAlogeHS was estimated from the Gifu sample of 5000 normal newborns. Then, using a Monte Carlo simulation as described previously for KD^12^, ^13^, ^27^ 100 million observations were generated from each of these estimated distributions and each test was applied to each sample point. The false positive rate of each BVNL test was then calculated as the percentage of points that were test‐positive. These calculated false positive rate were then compared to the theoretically expected *fp* = 10^−8^. Furthermore, the competing univariate threshold tests were compared to the BVNL‐based test.

## 3 RESULTS

### 3.1 BVNL results for normal newborns and MPSI patients

**Figure 1:**
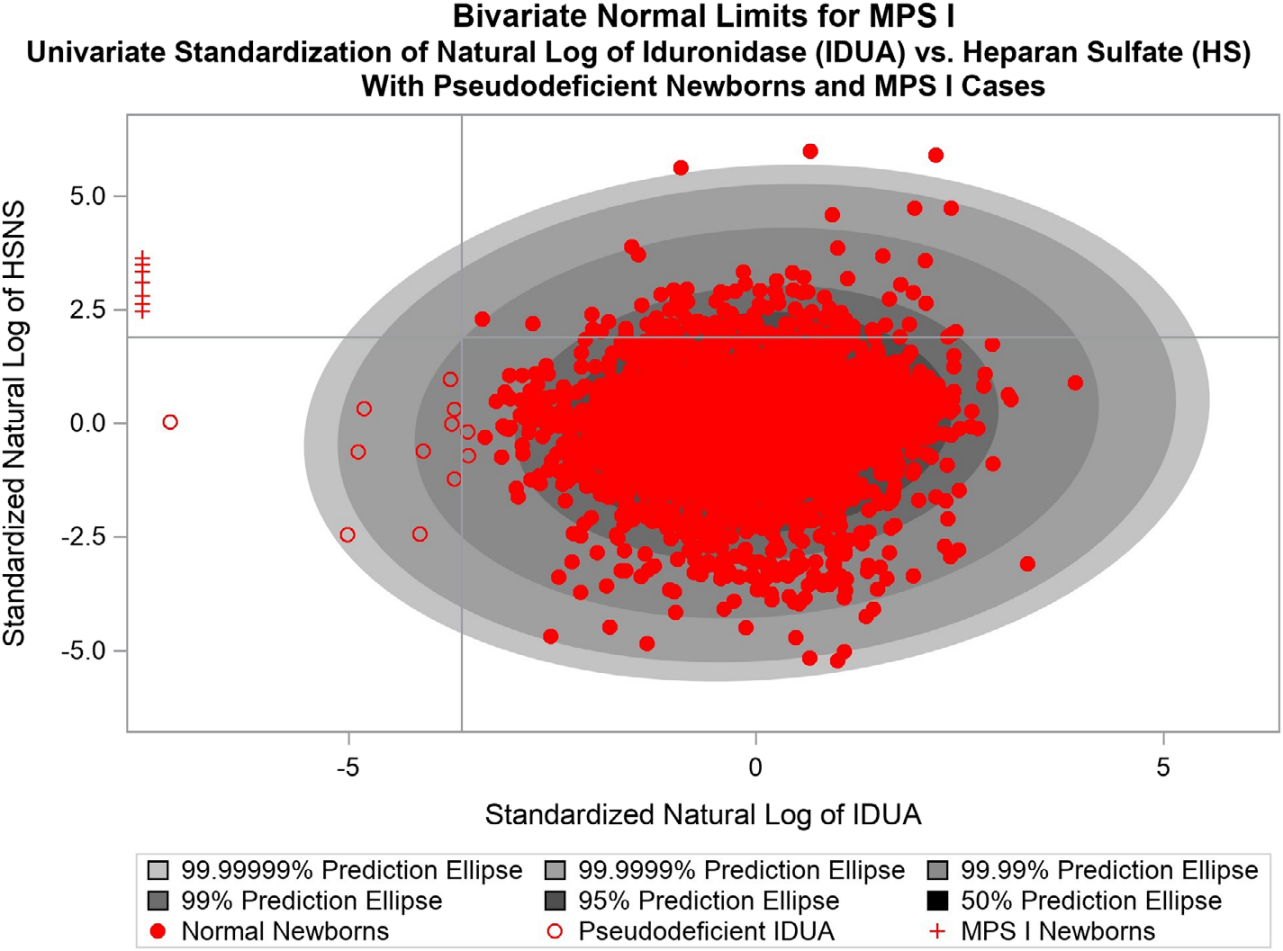
Illustration of the BVNL based on alpha‐L‐iduronidase enzyme activity (IDUA) and heparan sulfate and its application to 5000 normal newborns' and seven confirmed mucopolysaccharidosis type I (MPSI) patient's dried blood spots data. All data points from confirmed MPSI cases (red crosses) fall in the high‐risk region, while pseudo‐deficient cases are below the thresholds for standardized log_e_(HS) and therefore are correctly identified by the BVNL‐HS test

Figure 1 presents the results of simultaneous determination of the values of IDUA enzyme activities and HS measures for the 5000 normal newborns and the seven affected MPSI patients. The outer‐most ellipse is the (1‐10^−7^)100% prediction ellipse. The scatter of points conforms fairly well to the expected elliptical pattern under our working assumption of bivariate normality. This together with the approximate straight line pattern observed in the normal probability plots (not shown) for log_e_(IDUA) and log_e_(HS) provides evidence that our assumption that log_e_(IDUA) and log_e_(HS) have a bivariate normal distribution is valid. All of the 5000 newborns who were still disease free after approximately 2 years of follow‐up tested as negative by this BVNL‐HS NBS tool, including the 12 pseudo deficient newborns who were falsely predicted to be disease positive by the Gifu NBS protocol, which was based upon the univariate analysis of IDUA. Thus, the BVNL‐HS tool had 100% specificity and a 0% false positive rate. All of the seven confirmed MPSI cases were classified as test positive by the BVNL‐HS NBS tool. Therefore, this tool had 100% sensitivity and a 0% false negative rate.

### 3.2 Comparison of BVNL and univariate NBS tools

As shown in Table 1, while all three alternative tools displayed 100% sensitivity in identifying the seven confirmed MPSI cases, the PPV estimates for IDUA (0.50%), HS (0.17%), and IDUA/HS (70.27%) were substantially lower than that of the BVNL‐HS tool (99.9%). Note that while the IDUA/HS and BVNL‐HS use the same univariate thresholds because the BVNL‐HS tool additionally requires observations to fall outside of the (1‐10^−7^)100% prediction ellipse, and the estimated improvement in PPV is a natural consequence.

**Table 1:** Accuracy of bivariate normal limits (BVNL) and alternative alpha‐L‐iduronidase enzyme activity (IDUA) or glycosaminoglycan‐based newborn screening (NBS) tests for mucopolysaccharidosis type I

NBS test	Sensitivity%denominator	False neg.%#false neg.	Specificity%denominator	False pos.%#false pos.	PPV%estimator^a^
BVNL‐HS	100	0	100	0	99.90
	7	0	5000	0	
IDUA alone	100	0	99.80	0.20	0.50
	7	0	5000	10	
HS alone	100	0	99.42	0.58	0.17
	7	0	5000	29	
IDUA/HS	100	0	100	0	70.27
	7	0	5000	0	

a Positive predictive values (PPVs) were calculated by substituting sample estimates of sensitivity, a prevalence of 10^−5^ and estimates of false positive rate from the data, when false positive tests were observed (IDUA and heparan sulfate [HS] alone); or the false positive rate expected under bivariate normality when not (BVNL‐HS and IDUA/HS) and using the (1‐10^−7^)100% prediction ellipse where applicable (BVNL‐HS). The expected false positive rate of tests that were based on joint application of univariate thresholds was determined assuming that the two biomarkers involved were statistically independent. This assumption was supported as approximately valid by low correlations between IDUA and HS (*r* = 0.0934).

The results of our simulation study are shown in Table 2. PPV is again much higher in the BVNL‐HS tool (99.9%) than IDUA (6.25%), HS (0.034%), or IDUA/HS (84.96%).

**Table 2:** False positive rates and positive predictive values (PPVs) calculated from estimated sensitivities, a prevalence of 10^−5^ and normal newborn data simulated from bivariate normal distributions (n = 10^8^)

NBS test	Number of false positive test results	False positive%	PPV%
BVNL‐HS	1	10^−8^ × 100	99.90
IDUA alone	15 055	0.015	6.25
HS alone	2 889 334	2.889	0.034
IDUA, HS	177	0.000177	84.96

Abbreviations: BVNL, bivariate normal limits; HS, heparan sulfate; IDUA, alpha‐L‐iduronidase enzyme activity; NBS, newborn screening.

Taken together, the results above demonstrate that the BVNL‐HS test yields an estimated sensitivity of 100%, with expected specificity of 99.999999%, false positive rate of 10^−8^ (ie, 1 false positive test among 100 million newborns tested), and an estimated PPV of 99.9.

## 4 DISCUSSION

Each infant destined to be either afflicted with MPSI symptoms or to be spared from developing early symptoms despite low IDUA enzyme activity was accurately identified respectively as a case or noncase based upon their newborn DBS BVNL test result (Figure 1). These results suggest that the success of BVNL‐based tests as NBS tools is not confined to KD.^13^ It may, in fact, be generalizable not only to MPSI, but also to other screened conditions. However, a single test is performed usually on DBS used for NBS.^10^, ^11^, ^17^, ^18^ Determining second tier NBS biomarkers would require both a change in screening practice and additional expense, especially in countries with high birth rates or large numbers of newborns.

This study is limited by the small sample size of MPSI patients. There were only seven early‐onset Hurler patients whose newborn DBS were available for retrospective analyses. Since the incidence of MPSI has been approximated to be around 1/100 000 live births,^1^, ^18^, ^25^, ^26^ it was not unexpected that no new case emerged among the prospectively screened 5000 Japanese newborns (Figure 1). An earlier study showed elevated GAGs in DBS from 11 cases of MPS1.^28^ However, the current experiments extend that study and employ bivariate analyses.

If further prospective testing confirms the high PPV suggested here (99.9%), MPSI NBS will be improved significantly. The PPV suggested by this study is considerably higher than those reported in earlier pilot studies.^1^, ^16^, ^17^, ^19^ This PPV was estimated from a previously reported prevalence rate, the observed sensitivity, and a target false positive rate. The Monte Carlo simulation verified that this target false positive rate was achieved by the BVNL tool and suggests that these results could indeed be duplicated in a prospective study.

Two differences were noted in comparing the application of the BVNL approach to MPSI to its earlier application to KD.^13^ First, all seven of the MPSI cases had IDUA measurements that were at the limit of detection (Figure 1). This could conceivably suggest that univariate analysis of the DBS enzyme assay would have sufficed. However, the 12 pseudo‐deficiencies that represented false positive screens after univariate enzyme analyses of the 5000 DBS from the Japanese prefecture were only resolved correctly using the BVNL approach.

The MPSI cases being at the limit of enzyme detection (Figure 1) very likely relates to the need to cautiously define thresholds for NBS biomarkers. This need has been described in general with lysosomal storage disorders (LSDs)^19^, ^29^ and more specifically with GAG levels in MPS disorders.^4^, ^30^ In terms of the bivariate elliptical plots presented here for MPSI (Figure 1) and earlier for KD,^13^ the choice of a low threshold for enzyme level determined as a first tier NBS test would result in an extremely sensitive screening tool; a univariate test with a low threshold would presumably ensure the absence of false negative screens at the possible expense of increased false positive screens. Eliminating false negative results is a critical goal of NBS, and low‐threshold univariate tests are indeed the standard practice among screening programs, consistent with the urgent, ethically motivated need to identify every infant with a potentially treatable illness^19^, ^29^, ^31^, ^32^.

However, the choice of low (and therefore sensitive) enzyme thresholds is partially responsible for the lack of an acceptable balance between false positive and negative screens in NBS, as reported for the two disorders to which BVNL diagnostic tools have now been applied: KD^13^, ^33^ and MPSI.^14^, ^15^, ^16^, ^17^, ^18^, ^19^ There is also ethically motivated urgency to reduce high false positive rates.^32^ Using more aggressive thresholds to reduce false positive screens in a univariate test may, however, increase false negative screens. By combining BVNL ellipses with low‐enzyme and high‐GAG thresholds, it is possible to reduce false positives and false negatives simultaneously, as with previous BVNL application to KD.^13^

The second difference observed between the KD and MPSI BVNL tools was the presence of outliers only in the MPSI BVNL ellipse. Thus, in Figure 1, the measures of HS from three newborn DBS are discernible above the upper limit of the outer‐most ellipse. The reason for the emergence of these outliers is not currently known. It is possible that the outliers appear due in part to the significantly larger number of normal newborn DBS used to generate the BVNL plots (5000) as compared to the plots for KD (166).^13^ It is also possible that the acknowledged difficulty inherent to assays of GAGs on DBS^4^ caused more variability in the MPSI data.

These results present proof of principle that a BVNL‐based approach can be applied to NBS for MPSI. The BVNL approach to NBS is being considered by several US states currently screening for KD and MPSI, and it is in use by the Gifu screening program. While implementation requires access to a statistical program, the R program that we employ^22^, ^24^ is widely available. Furthermore, with our enabled estimation of a PPV of 99.9%, and our selection of *fp* = 10^−8^, approximately one false positive screen in expected after screening one hundred million normal newborns. Thus, in light of the expected US birth rate of at most 4 million annually,^34^ it is predicted that one false positive would emerge every 25 years if the BVNL‐based test was applied to every US newborn. This would represent a remarkable improvement in NBS for MPSI.^14^, ^15^, ^16^, ^17^, ^18^, ^19^

No new case was identified among the 5000 prospective screens from the Gifu prefecture. New York State recently reported the results of 65 000 LSDs newborn screens.^19^ No early‐onset (Hurler) patients were identified in New York, and indeed among all the LSD screens performed the only positives were predicted to have late‐onset phenotypes.^19^ Based upon these NY results, the need to more accurately stratify infants identified at NBS based upon their anticipated ages of onset has been emphasized.^35^

The results presented here indicate the potential of the BVNL‐based approach to MPSI to clearly predict early‐onset of symptoms, and to enable identification of newborns who, notwithstanding positive screens, are not at risk for early symptoms. This would further the important goals of accurate age of onset‐based stratification^35^ based upon data available from newborn DBS, and of the earliest possible treatment of infants who are destined to develop early symptoms of MPSI.^5^, ^6^, ^7^

## CONFLICT OF INTEREST

The authors declare no potential conflict of interest.

## AUTHOR CONTRIBUTIONS

T.J.L.: Study design, methodology, investigation, oversight of study activities, funding acquisition, data curation, formal analysis, drafting and editing of manuscript. K.J.: Study design, methodology, investigation, resources, data curation, formal analysis, drafting and editing of manuscript. A.B.: Study design, methodology, investigation, resources, data curation, drafting and editing of manuscript. R.C.: Study design, methodology, investigation, funding acquisition, data curation, formal analysis, drafting and editing of manuscript. M.S.: Study design, methodology, investigation, resources, editing of manuscript. K.O.: Study design, methodology, investigation, resources, editing of manuscript. T.F.: Study design, methodology, investigation, resources, editing of manuscript. H.K.: Study design, methodology, investigation, resources, editing of manuscript. S.Y.: study design, methodology, investigation, resources, editing of manuscript. S.T.: Study design, methodology, investigation, oversight of study activities, data curation, drafting and editing of manuscript.

## ETHICS STATEMENT

This study was approved by the University at Buffalo Institutional Review Board.

## Data Availability

The de‐identified data are maintained in the Longitudinal Pediatric Data Resource (LPDR) of the Newborn Screening Translation Research Network (NBSTRN).
